# The effect of different timings of protein supplementation on variable outcomes in hemodialysis patients: a randomized clinical trial

**DOI:** 10.1007/s10157-025-02626-7

**Published:** 2025-01-18

**Authors:** Mohamed Mamdouh Elsayed, Amr Mohamed Elkazaz

**Affiliations:** 1 Nephrology and Internal Medicine Department, Faculty of Medicine, Alexandria University, Alkhartoom Square, El Azareeta, Alexandria, 21131 Egypt; 2 Internal Medicine Department, El Qabbary General Hospital, Ministry of Health, Alexandria, Egypt

**Keywords:** Protein supplement, ONS, Timing, Hemodialysis

## Abstract

**Background:**

Oral nutritional supplements (ONS) are commonly prescribed to provide protein and energy to hemodialysis (HD) patients. There is a debate about the appropriate timing to administer ONS. We aimed to study the effect of different timings of ONS on variable outcomes in HD patients.

**Methods:**

This research is a prospective, randomized, multicentric clinical trial (RCT) that included 120 patients on regular HD. Patients were allocated to receive ONS (25 gm protein powder/HD session) for 8 weeks either predialytic (1 h before the start of the session), intradialytic (2 h after the start of the session), or interdialytic (on non-dialysis days). Laboratory parameters, blood pressure (BP), dialysis adequacy, and nutritional parameters were assessed during the study.

**Results:**

At study end, BP at the end of HD dropped significantly in the intradialytic group compared to the other groups (p < 0.001). Serum albumin improved significantly in the predialytic (p < 0.001) and intradialytic (p = 0.039) groups. The mean subjective global assessment score increased significantly in the interdialytic group (p = 0.040). The Kt/V and urea reduction ratio decreased significantly only in the intradialytic group (*p* value < 0.001 and 0.001). Serum sodium, potassium, phosphorus, cholesterol, triglycerides, and adverse events did not significantly differ between the different groups.

**Conclusions:**

Predialytic ONS supplementation is a favorable option due to improved serum albumin with minimal effects on hemodynamics and dialysis adequacy compared to intradialytic and interdialytic supplementation.

**Clinical Trials registration:**

ClinicalTrials.gov NCT05953636.

First registration date: 1/07/2023.

## Introduction

The prevalence of chronic kidney disease (CKD) is predicted to rise over the next few decades, accompanied by a progressive increase in end stage renal disease (ESRD) incidence [1]. Malnutrition is a common health problem in patients on regular hemodialysis (HD) and is strongly linked to increased morbidity and mortality [2]. Prompt diagnosis and management of malnutrition in HD patients is essential to avoid its harmful consequences [3].

Oral nutritional supplements (ONS) are commonly prescribed by nephrologists to provide protein and energy to malnourished HD patients who cannot meet their nutritional requirements from a regular diet, taking into consideration the possible rise in serum phosphorus and potassium [4, 5]. Many studies have demonstrated that ONS consumption is associated with improvements in protein energy wasting, serum albumin, quality of life, and clinical outcomes in dialysis patients [6–9].

There is much controversy about the appropriate timing for administering ONS in the dialysis population [10]. The administration of ONS during HD sessions (intradialytic) is preferred by some physicians due to better compliance and closer supervision of patients. However, there are some concerns about the increased risk of intradialytic hypotension (IDH), gastrointestinal upset, and dialysis inadequacy with intradialytic feeding [11–13]. The second option is to administer ONS in dialysis units before the start of the HD session (predialytic) to ensure compliance, which was associated with better dialysis adequacy than intradialytic supplementation in a recent study [14]. ONS can also be given on non-dialysis days (interdialytic) to avoid hemodynamic instability during HD sessions, but its efficacy and safety have not been well studied. To date, there is no consensus about the best time to supply ONS to dialysis patients. Therefore, in this study, we aimed to study the effect of different timings (interdialytic, predialytic, and intradialytic) of protein supplementation on variable outcomes in HD patients.

## Materials and methods

### Study participants and design

This research is a prospective, randomized multicentric clinical trial which enrolled 120 patients from different dialysis units in Alexandria. We included ESRD patients who were maintained on regular HD (3 times/week, 4 h/HD session for more than 6 months) and aged ≥ 18 years. The block randomization method was used to randomly assign patients to receive oral protein nutritional supplement (ONS) [Fresubin protein powder (Fresenius Kabi Deutchland, Bad Homburg, Germany) 25 gm (5 scoops) per HD session] for 8 weeks: predialytic (1 h before the start of the session), intradialytic (2 h after the start of the session), or interdialytic (on non-dialysis days, three times/week at the exact time of their dialysis session either morning or evening). Fresubin is a whey protein powder supplement (fiber free). Each 5 gm (1 scoop) of fresubin provides 18 kcal of energy, composed mainly of protein (4.4 gm, 97% of total energy), with trace amounts of fat (0.05 gm, 2% of total energy), carbohydrates (≤ 0.05 gm, ≤ 1% of total energy) and minerals (sodium 27.5 mg, potassium 60 mg, calcium 3 mg and phosphorus 12 mg). Fresenius Kabi company provided us with the supplement for free without any cost. Allocation concealment was ensured using a sealed closed envelope randomization technique, and every patient was given an identification code. Standard dietary recommendations were provided to all patients in the three groups to optimize dietary nutritional intake according to the Kidney Disease Outcomes Quality Initiative (KDOQI) guidelines with close follow up during study period. Patients’ dietary records included all nutritional information and were submitted every two weeks. We used the 3-day food record to assess the dietary intake. The exclusion criteria were liver failure, pregnancy, malignancy, an allergy to any ingredient in the nutritional supplements, or receiving nutritional supplementation within the last two months. The trial was registered on Clinicaltrials.gov (NCT05953636) (1/07/2023).

### Methods

All study participants had a thorough history taking, with an emphasis on demographic data, cause of ESRD, duration of dialysis, modality of dialysis, and comorbidities. A thorough physical examination was conducted. Blood pressure (BP) was measured before the session and then every hour (reported BP is the mean BP for the 24 HD sessions over the 8 week study period). Assessment of dialysis adequacy was performed at baseline and after 8 weeks using the single-pool Kt/V Daugirdas formula (second generation) [15]. All participants were asked at the end of the session if they experienced nausea, vomiting, cramps or hypotensive episodes. IDH was defined as a decrease in systolic BP of ≥ 20 mmHg associated with symptoms.

The subjective global assessment (SGA) scale was used to assess nutritional status at the beginning and at the end of the study. The SGA questionnaire includes two components: medical history and physical examination. The medical history section includes changes in weight, nutritional consumption, gastrointestinal problems, functional capacity, and disease and comorbidity status. Muscle wasting, edema, and loss of subcutaneous fat are all examined in the physical component. According to their overall SGA scores, patients were divided into 3 groups (7–13: normal), (14–23: mildly to moderately malnourished) and (24–35: severely malnourished) [16]. The study investigators who were trained on how to calculate the SGA by the hospital nutritional specialist, conducted the assessment following the dialysis session.

Laboratory investigations including blood urea, creatinine, sodium (Na), potassium (K), calcium (Ca), phosphorus (Ph), total cholesterol, triglycerides, intact parathormone (iPTH), albumin, complete blood count, and C-reactive protein (CRP) were measured at baseline and at study end.

### Statistical analysis

The data were fed to the computer and analyzed using the IBM SPSS software package version 20.0. **(**Armonk, NY: IBM Corp**)**. Categorical data were represented as numbers and percentages. The Chi-square test was applied to compare between three groups. Alternatively, Fisher Exact test was applied when more than 20% of the cells have expected count less than 5. To analyze the significance between two periods, the Marginal Homogeneity Test was used. For continuous data, they were tested for normality by the Shapiro–Wilk test. Quantitative data were expressed as mean and standard deviation. For normally distributed quantitative variables, One way ANOVA test was used for comparing the three studied groups while Paired t-test was used to compare between two periods. On the other hand, for not normally distributed quantitative variables, the Kruskal Wallis test was used to compare three groups, while the Wilcoxon signed ranks test was used to compare between two periods. Significance of the obtained results was judged at the 5% level. Sample size was calculated using Power Analysis and Sample Size Software (PASS 2020) “NCSS, LLC. Kaysville, Utah, USA, ncss.com/software/pass”. The minimal total hypothesized sample size of 120 eligible HD patients (40 per group) is needed to study the effect of different timings of protein supplementation on variable outcomes in HD patients; taking into consideration an assumed effect size (Minimally Clinically Important Difference) of 30%, 95% level of confidence, compliance ratio (1:1), and power of 80% using Chi square-test [14].

## Results

### Patient characteristics

**Fig. 1:**
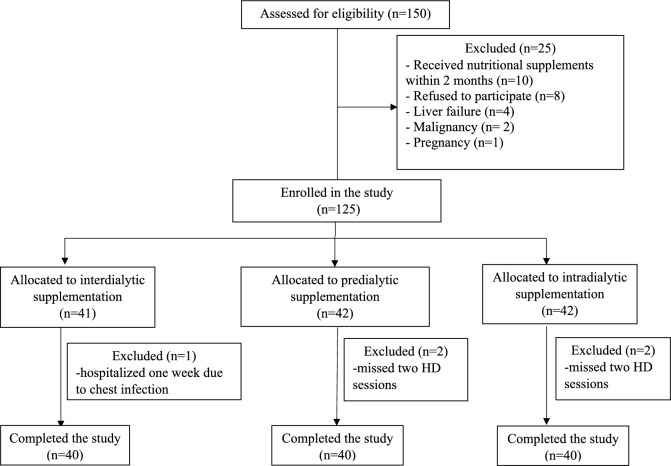
Patient flow chart

One hundred and fifty patients were assessed to participate in the study and 30 patients were excluded. Therefore, we enrolled a total of 120 HD patients. Following randomization, 40 patients in each group were given ONS either interdialytic, predialytic, or intradialytic for 8 weeks (Fig. 1). Table 1 displays the baseline characteristics of the patients. At baseline, age, gender, body mass index (BMI), ESRD etiology, comorbidities, HD duration and prescription, vascular access, different laboratory parameters, and daily energy (DEI) and protein intake (DPI) did not significantly differ between the different groups.

**Table 1:** Baseline characteristics of the study groups

	Interdialytic group (n = 40)	Predialytic group (n = 40)	Intradialytic group (n = 40)	*p*
Age (years)	53.76 ± 12.44	51.32 ± 10.52	52.84 ± 12.34	0.762
Sex				
Male	16 (40.0%)	16 (40.0%)	17 (42.5%)	0.946
Female	24 (60.0%)	24 (60.0%)	23 (57.5%)	
BMI (kg/m^2^)	25.76 ± 4.03	25.23 ± 4.94	25.48 ± 4.31	0.915
Smoking	8 (20.0%)	8 (20.0%)	10 (25.0%)	0.924
Duration of HD (years)	5.60 ± 4.35	3.88 ± 3.06	4.68 ± 4.40	0.225
HD prescription				
Blood flow rate (ml/min) (QB)	304.8 ± 34.66	328.0 ± 25.33	320.0 ± 25.0	0.443
Dialysate flow (ml/hr) (QD)	500.0 ± 0.00	524.0 ± 83.07	500.0 ± 0.00	0.132
UF volume (L/session)	3.15 ± 0.63	2.94 ± 0.78	2.78 ± 0.91	0.249
KT/V	1.34 ± 0.20	1.34 ± 0.29	1.42 ± 0.21	0.376
Vascular access				
AVF	32 (80.0%)	37 (92.5%)	35 (87.5%)	0.619
Catheter	6 (15.0%)	3 (7.5%)	5 (12.5%)	
Graft	2 (5.0%)	0 (0.0%)	0 (0.0%)	
Cause of ESRD				
Hypertension	16 (40.0%)	14 (35.0%)	18 (45.0%)	0.681
DM	11 (27.5%)	11 (27.5%)	10 (25.0%)	0.947
Glomerulonephritis (GN)	9 (22.5%)	11 (27.5%)	5 (12.5%)	0.458
ADPCKD	0 (0.0%)	2 (5.0%)	0 (0.0%)	1.000
Others	4 (10.0%)	2 (5.0%)	7 (17.5%)	0.304
Comorbidities				
Hypertension	24 (60.0%)	30 (75.0%)	30 (75.0%)	0.200
Heart failure	8 (20.0%)	8 (20.0%)	6 (15.0%)	0.927
IHD	15 (37.5%)	19 (47.5%)	26 (65.0%)	0.225
Total cholesterol (mg/dl)	193.6 ± 43.15	177.3 ± 29.27	187.1 ± 41.72	0.325
Serum triglycerides (mg/dl)	153.8 ± 52.76	130.3 ± 35.13	144.8 ± 61.59	0.156
Hemoglobin (g/dl)	10.20 ± 1.31	10.68 ± 1.66	10.46 ± 1.89	0.587
Serum albumin (g/dl)	3.60 ± 0.54	3.86 ± 0.49	3.77 ± 0.45	0.171
Serum calcium (mg/dl)	9.09 ± 1.21	9.43 ± 1.42	9.37 ± 9.37	0.630
Serum phosphorus (mg/dl)	5.56 ± 1.73	6.44 ± 3.21	5.12 ± 2.36	0.176
Serum PTH (pg/ml)	697.8 ± 749.3	706.1 ± 624.4	612.0 ± 451.6	0.968
Serum sodium (mEq/L)	137.0 ± 3.73	138.0 ± 3.70	138.8 ± 4.58	0.304
Serum potassium (mEq/L)	5.28 ± 0.83	5.58 ± 0.87	5.62 ± 0.95	0.335
CRP (mg/l)	9.34 ± 17.20	6.64 ± 5.93	7.44 ± 5.80	0.804
Daily energy intake (kcal/kg/day)	25.17 ± 3.35	25.67 ± 3.08	25.43 ± 3.26	0.932
Daily protein intake (gm/kg/day)	0.88 ± 0.45	0.92 ± 0.34	0.90 ± 0.53	0.878

*p*: p value for comparing between the three studied groups

Data were expressed as Mean ± Standard deviation (SD), or absolute numbers as appropriate.

*ADPCKD* autosomal dominant polycystic kidney disease, *AVF* arteriovenous fistula, *BMI* body mass index, *CRP* C reactive protein, *DM* diabetes mellitus, *HD* hemodialysis, *IHD* ischemic heart disease, *Kt/V* measuring dialysis adequacy, *PTH* parathyroid hormone, *UF* ultrafiltration.

### Effect on blood pressure

At baseline, there was no significant difference in the MAP between the study groups. At the end of the study, the MAP before HD session increased significantly compared to that at the beginning of the study in the predialytic (p < 0.001) and intradialytic (p = 0.012) groups but not significantly in the interdialytic group (p = 0.505). After 8 weeks of supplementation, the MAP at the end of the session (4 h) decreased significantly in the intradialytic group (p < 0.001) but increased significantly in the predialytic (p < 0.001) and non-significantly in the interdialytic (p = 0.911) groups compared to the baseline readings in each group. Additionally, at the end of the study, the MAP at the end of the session in the intradialytic group was significantly lower than that in the predialytic and interdialytic groups (p < 0.001) (Table 2).

**Table 2:** M**ean** arterial blood pressure readings during HD in study groups

MAP (mmHg)	Interdialytic group (n = 40)			Predialytic group (n = 40)			Intradialytic group (n = 40)			Comparison bet. gps. at baseline	Comparison bet. gps. at week 8
	Baseline	Week 8	*p*_*0*_	Baseline	Week 8	*p*_*0*_	Baseline	Week 8	*p*_*0*_	*p*_*1*_	*p*_*2*_
Pre-HD	111.2 ± 11.46	113.4 ± 12.37	0.505	106.5 ± 12.37	110.8 ± 12.77	< 0.001^*^	107.8 ± 10.28	110.6 ± 10.83	0.012^*^	0.337	0.653
After 1 h. HD	109.3 ± 11.78	110.8 ± 12.14	0.668	110.3 ± 15.37	110.0 ± 13.70	0.796	109.0 ± 11.58	109.4 ± 11.43	0.499	0.934	0.919
After 2 h. HD	107.5 ± 13.48	106.8 ± 13.27	0.864	104.3 ± 16.04	105.7 ± 14.71	0.225	106.3 ± 12.76	105.9 ± 11.67	0.737	0.728	0.953
After 3 h. HD	102.7 ± 13.47	103.0 ± 13.97	0.947	96.56 ± 14.54	101.1 ± 14.48	0.002^*^	99.28 ± 12.80	101.4 ± 13.40	0.083	0.288	0.884
After 4 h. HD	98.28 ± 14.35	98.76 ± 14.45	0.911	94.36 ± 10.65	102.8 ± 8.54	< 0.001^*^	91.92 ± 14.04	76.16 ± 8.69	< 0.001^*^	0.288	< 0.001^*^

Data were expressed in Mean ± SD.

*p*_*0*_: p value for comparing between baseline and week 8 in each group

*p*_*1*_: p value for comparison bet. groups at baseline

*p*_*2*_: p value for for comparison bet. groups at week 8

*Statistically significant at p ≤ 0.05

*MAP* mean arterial pressure

### Effect on nutritional parameters and dialysis adequacy

At the end of the study, the Kt/V and URR decreased significantly in the intradialytic group with *p* values of < 0.001 and 0.001, respectively, but not significantly in the predialytic or interdialytic group.

Regarding nutritional parameters, serum albumin improved significantly in the predialytic (p < 0.001), and intradialytic (p = 0.039) groups but decreased non-significantly in the interdialytic group (p = 0.243), with a significant difference among the three groups (p < 0.001). BMI, DEI and DPI did not significantly differ among the different groups. At baseline, malnutrition was present in 27.5%, 27.5%, 30% of patients in the interdialytic, predialytic and intradialytic groups, respectively. The mean SGA increased significantly in the interdialytic group (p = 0.040) and decreased non-significantly in both the predialytic and intradialytic groups, with *p* values of 0.755 and 0.328, respectively (Table 3).

**Table 3D:** **ialysis** adequacy and nutritional parameters at baseline and study end in study groups

	Interdialytic group (n = 40)			Predialytic group (n = 40)			Intradialytic group (n = 40)			Comparison bet. grps. at week 8
	Baseline	Week 8	*p*_*0*_	Baseline	Week 8	*p*_*0*_	Baseline	Week 8	*p*_*0*_	*p*
KT/V Daugridas	1.34 ± 0.20	1.33 ± 0.17	0.700	1.34 ± 0.29	1.27 ± 0.21	0.184	1.42 ± 0.21	1.30 ± 0.20	< 0.001^*^	0.547
URR	67.55 ± 8.01	67.29 ± 7.15	0.865	67.47 ± 12.22	64.72 ± 8.58	0.253	69.99 ± 8.79	66.16 ± 8.25	0.001^*^	0.527
Serum albumin (g/dl)	3.60 ± 0.54	3.51 ± 0.53	0.243	3.86 ± 0.49	4.18 ± 0.40	< 0.001^*^	3.77 ± 0.45	3.95 ± 0.44	0.039^*^	< 0.001^*^
Post dialysis weight (kg)	75.91 ± 16.22	75.54 ± 17.0	0.550	73.12 ± 14.69	73.04 ± 15.03	0.717	74.14 ± 15.72	74.02 ± 11.04	0.472	0.373
BMI (kg/m^2^)	25.76 ± 4.03	25.35 ± 4.06	0.069	25.23 ± 4.94	25.25 ± 5.0	0.800	25.48 ± 4.31	25.39 ± 4.45	0.460	0.994
Daily energy intake (kcal/kg/day)	25.17 ± 3.35	25.10 ± 3.22	0.885	25.67 ± 3.08	25.74 ± 3.17	0.912	25.43 ± 3.26	25.55 ± 3.18	0.893	0.910
Daily protein intake (gm/kg/day)	0.88 ± 0.45	0.86 ± 0.47	0.945	0.92 ± 0.34	0.93 ± 0.41	0.923	0.90 ± 0.53	0.91 ± 0.57	0.985	0.945
SGA (points)										
Normal well nourished	29 (72.5%)	26 (65.0%)	0.317	29 (72.5%)	30 (75.0%)	0.157	28 (70.0%)	29 (72.5%)	0.317	0.865
Mild to moderate MN	9 (22.5%)	12 (30.0%)		10 (25.0%)	10 (25.0%)		10 (25.0%)	10 (25.0%)		
Severe MN	2 (5.0%)	2 (5.0%)		1 (2.5%)	0 (0.0%)		2 (5.0%)	1 (2.5%)		
Mean ± SD	12.20 ± 4.07	13.56 ± 4.61	0.040^*^	12.32 ± 4.11	11.76 ± 2.57	0.755	12.72 ± 4.08	12.32 ± 4.67	0.328	0.277

Data were expressed in Mean ± SD, or absolute numbers as appropriate.

^*t*^*p*_*0*_: p value for comparing between baseline and week 8 in each group

*p*: p value for comparison bet. 3 groups at week 8

*Statistically significant at p ≤ 0.05

*BMI* body mass index, *MN* malnutrition, *SGA* subjective global assessment, *URR* urea reduction ratio.

### Effects on laboratory parameters & adverse events

Total cholesterol, triglycerides, calcium, phosphorus, PTH, sodium, potassium, hemoglobin and CRP did not significantly differ between the different groups after supplementation. A comparison of the results for each group revealed that hemoglobin decreased significantly in the intradialytic (p = 0.008) and predialytic (p = 0.017) groups. Serum PTH increased significantly in the predialytic (p < 0.001) group. Serum sodium decreased significantly in the interdialytic, predialytic and intradialytic groups with *p* values of 0.002, 0.006 and 0.002, respectively (Table 4).

**Table 4:** L**ab** or **at** or **y** parameters at baseline and study end in study groups

	Interdialytic group (n = 40)			Predialytic group (n = 40)			Intradialytic group (n = 40)			Comparison bet. grps at week 8
	Baseline	Week 8	*p*_*0*_	Baseline	Week 8	*p*_*0*_	Baseline	Week 8	*p*_*0*_	*P*
Total cholesterol (mg/dl)	193.6 ± 43.15	196.0 ± 37.88	0.603	177.3 ± 29.27	182.6 ± 30.49	0.053	187.1 ± 41.72	183.6 ± 38.97	0.399	0.343
Serum triglycerides (mg/dl)	153.8 ± 52.76	153.6 ± 50.10	0.587	130.3 ± 35.13	142.3 ± 35.08	0.394	144.8 ± 61.59	137.7 ± 57.14	0.209	0.818
Hemoglobin (g/dl)	10.20 ± 1.31	10.16 ± 1.58	0.726	10.68 ± 1.66	10.09 ± 1.84	0.017^*^	10.46 ± 1.89	9.67 ± 1.80	0.008^*^	0.569
Serum calcium (mg/dl)	9.09 ± 1.21	9.20 ± 1.03	0.502	9.43 ± 1.42	9.53 ± 1.10	0.512	9.37 ± 1.36	9.54 ± 1.10	0.679	0.466
Serum phosphorus (mg/dl)	5.56 ± 1.73	5.14 ± 1.51	0.191	6.44 ± 3.21	6.13 ± 2.14	0.373	5.12 ± 2.36	5.22 ± 1.44	0.824	0.084
Serum PTH (pg/ml)	697.8 ± 749.3	703.2 ± 758.8	0.925	706.1 ± 624.4	727.0 ± 677.8	< 0.001^*^	612.0 ± 451.6	705.9 ± 570.7	0.139	0.263
Serum sodium (mEq/L)	137.0 ± 3.73	134.9 ± 3.09	0.002^*^	138.0 ± 3.70	135.2 ± 2.60	0.006^*^	138.8 ± 4.58	134.8 ± 4.20	0.002^*^	0.877
Serum potassium (mEq/L)	5.28 ± 0.83	4.98 ± 0.85	0.065	5.58 ± 0.87	5.33 ± 1.0	0.166	5.62 ± 0.95	5.38 ± 0.91	0.140	0.267
CRP (mg/l)	9.34 ± 17.20	9.37 ± 12.06	0.113	6.64 ± 5.93	8.40 ± 8.85	0.191	7.44 ± 5.80	6.78 ± 3.19	0.557	0.629

Data were expressed in Mean ± SD

*p*_*0*_: p value for comparing between baseline and week 8 in each group

*p*: p value for Comparison between groups at week 8

*Statistically significant at p ≤ 0.05

Regarding adverse events, diarrhea, nausea, and IDH episodes were reported in the three groups without a significant difference with *p* value of 1.000, 0.846 and 0.903 respectively.

## Discussion

HD patients who do not achieve their nutritional requirements are usually given ONS; however, there is no strong evidence about the best timing to offer these supplements. In the present study, we compared different timings and found that predialytic and intradialytic supplementation were associated with improvement in serum albumin levels. Additionally, intradialytic feeding was accompanied by more hemodynamic instability and dialysis inadequacy.

The problem is that most of the trials testing the appropriate timing of nutritional supplementation in the HD population have been conducted on a small number of patients who mostly received ONS during HD [17]. Very few studies have tested the concept of giving ONS either before HD, or on non-dialysis days. To our knowledge, we are the first to compare the three timings in the same study.

As illustrated above, we found that serum albumin improved significantly with predialytic and intradialytic supplementation. Additionally, the mean SGA increased significantly only in the interdialytic group and the DPI did not significantly differ between the different groups. These results revealed better and improved serum albumin levels with predialytic and intradialytic supplements than with interdialytic supplements. These findings could be due to better compliance and close observation for patients to consume supplements in HD units, while with interdialytic supplementation, we cannot assure high level of compliance. Hypoalbuminemia is considered an important marker for protein energy wasting (PEW) [18] and a predictor of mortality in HD patients [19]. Therefore, we should advocate increasing serum albumin to avoid these deleterious effects. Recent systematic reviews and meta-analyses [17, 20] revealed that ONS was associated with improvements in serum albumin, but the data about timing were conflicting. Some reported that non-intradialytic but not intradialytic supplementation improved serum albumin concentration [17]; however, others reported the opposite [11, 21]. The authors explained their results that in the non-intradialytic form, patients were given ONS daily rather than only thrice weekly in the intradialytic supplemented groups. Additionally, we found that BMI did not differ between the different timings and was not improved by ONS. However, other researchers [17, 20] revealed significant improvement with ONS consumption. Also, we did not find significant difference between different timings regarding DEI. To our knowledge, this association has not been tested before. However, many studies that reported increased serum albumin levels involved supplementations that provided around 200–300 kcal of energy in addition to protein.

In the present work, we found that with intradialytic supplementation, there was a significant decrease in BP toward the end of HD sessions, which was not present with predialytic and interdialytic supplements. This is usually explained by the redirection of blood to the splanchnic vessels after feeding, which causes this hemodynamic instability. Intradialytic hypotension is the main fear of intradialytic meals because of its close association with increased morbidity and mortality in HD patients [22]. Borzou et al., in their study that enrolled 48 HD patients, reported a significant drop in BP following a meal given at 1 h and 2 h during HD [23]. Other researchers reported a drop in relative blood volume (RBV) and BP following intradialytic meals [24].

We found a significant decrease in dialysis adequacy only with intradialytic supplementation. Many explanations have been implicated in pathogenesis, including the drop in BP and RBV with intradialytic feeding causing diminished urea clearance. Additionally, protein intake is associated with increased urea generation, which can affect Kt/V and URR. Another mechanism might be the method of measuring Kt/V. Muller-Delle et al. [25] evaluated Kt/V following an intradialytic meal and found a drop when using a UV absorbance system but not when using the ionic dialysance method. They attributed this difference between methods to an increase in UV absorbent solutes following meals. Many studies [5, 26] have reported reduced dialysis adequacy with intradialytic nutrition (meals). Rao et al. [14], in their prospective crossover study that included 72 patients maintained on twice weekly HD, compared the predialytic vs intradialytic administration of ONS (not a meal) for 2 consecutive weeks regarding dialysis adequacy, BP and urea removal. They found that predialytic nutrition was associated with better control of BP and better dialysis adequacy than intradialytic administration, which is in line with our findings. We differ from their study that our patients were on thrice weekly HD, we assessed the option to administer ONS on non-dialysis days (interdialytic). Our study was a 3 group RCT (not crossover), and we also tested the effect of different timings on nutritional and laboratory parameters.

Regarding adverse events, we found no significant difference in the reported adverse events (nausea, diarrhea and hypotensive episodes) between different timings. This is consistent with the findings of other studies [14, 27]. This finding does not contradict our finding of a significant drop in MAP with intradialytic supplementation because we defined intradialytic hypotension as a drop in systolic BP of ≥ 20 mmHg associated with symptoms. Based on this definition, we found that IDH episodes were comparable among different groups. However, other studies revealed more IDH episodes with intradialytic feeding [5].

Additionally, we did not find significant difference between different timings regarding serum phosphorus, potassium, CRP, and other laboratory parameters. To our knowledge, this association has not been tested before. This relation is important for clarifying whether if there is an increased risk of hyperkalemia or hyperphosphatemia with specific timing. Most of the published data focused on the effect of ONS in general (not timing) on serum phosphorus, potassium, and CRP and revealed no significant effect according to 2 recent systematic reviews [17, 20]. This is consistent with our findings, as we did not find significant changes inside each group at the end of the study. Also, we found that hemoglobin decreased significantly in the intradialytic and predialytic groups, and serum PTH increased in the predialytic (p < 0.001) group without a significant difference between the three groups. We do not have a clear explanation for these findings. Serum sodium decreased significantly in all groups, most probably due to our strict instructions for all patients to be very cautious with their sodium intake because the supplement contained Na already but there was no significant difference between different timings regarding sodium, hemoglobin, and PTH.

The strengths of our study include being the first study to test three possible timings (inter, pre, and intradialytic) to administer ONS to HD patients in the same study. Additionally, we assessed their effects on many outcomes, including BP, dialysis adequacy, nutritional status, laboratory parameters, and adverse events. Another point is that we administered ONS with the same composition (not a meal) to our patients to limit variability. Possible drawbacks of our study might be the supplementation period (8 weeks), and longer durations would have strengthened our findings. Additionally, we cannot ensure compliance in the interdialytic group which could explain the reduced effect of ONS on those patients. Also, we included only 120 patients from different HD centers but in the same city and the enrollment of more patients from other regions would have been more confirmatory to our results.

## Conclusion

Predialytic administration of ONS seems to be a favorable option in dialysis patients due to the improvement in serum albumin levels with minimal effects on hemodynamics and dialysis adequacy. Intradialytic timing was associated with more dialysis inefficiency and hemodynamic instability despite better nutrition. Administering ONS on non-dialysis days (interdialytic) was not also the favorable option as it failed to improve serum albumin, although it did not cause harm to patients. Larger RCTs with longer follow-up periods are needed to confirm these results.

## Data Availability

The datasets used and/or analyzed during the current study are available from the corresponding author on reasonable request.
